# Co-activation of the diaphragm and external intercostal muscles through an adaptive closed-loop respiratory pacing controller

**DOI:** 10.3389/fresc.2023.1199722

**Published:** 2023-07-07

**Authors:** Rabeya Zinnat Adury, Ricardo Siu, Ranu Jung

**Affiliations:** ^1^ Department of Applied Physiology and Kinesiology, University of Florida, Gainesville, FL, United States; ^2^ Department of Biomedical Engineering, Florida International University, Miami, FL, United States; ^3^ Department of Physical Medicine and Rehabilitation, Case Western Reserve University, Cleveland, OH, United States; ^4^ Department of Biomedical Engineering, The Institute for Integrative and Innovative Research (I^3^R), University of Arkansas, Fayetteville, AR, United States

**Keywords:** closed-loop system, ventilatory control system, stimulation, respiratory pacing, sighs, augmented breaths, stimulation-induced fatigue

## Abstract

**Introduction:**

Respiratory pacing is a promising alternative to traditional mechanical ventilation that has been shown to significantly increase the survival and quality of life after the neural control of the respiratory system has been compromised. However, current pacing approaches to achieve adequate ventilation tend to target only the diaphragm without pacing external intercostal muscles that are also activated during normal inspiration. Furthermore, the pacing paradigms do not allow for intermittent sighing, which carries an important physiological role. We hypothesized that simultaneous activation of the diaphragm and external intercostal muscles would improve the efficiency of respiratory pacing compared to diaphragm stimulation alone.

**Materials and Methods:**

We expanded an adaptive, closed-loop diaphragm pacing paradigm we had previously developed to include external intercostal muscle activation and sigh generation. We then investigated, using a rodent model for respiratory pacing, if simultaneous activation would delay the fatigability of the diaphragm during pacing and allow induction of appropriate sigh-like behavior in spontaneously breathing un-injured anesthetized rats (*n* = 8) with pacing electrodes implanted bilaterally in the diaphragm and external intercostal muscles, between 2nd and 3rd intercostal spaces.

**Results:**

With this novel pacing system, we show that fatigability of the diaphragm was lower when using combined muscle stimulation than diaphragm stimulation alone (*p* = 0.014) and that combined muscle stimulation was able to induce sighs with significantly higher tidal volumes compared to diaphragm stimulation alone (*p *= 0.014).

**Conclusion:**

Our findings demonstrate that simultaneous activation of the inspiratory muscles could be used as a suitable strategy to delay stimulation-induced diaphragmatic fatigue and to induce a sigh-like behavior that could improve respiratory health.

## Introduction

Following acute cervical cord injury, brainstem disease, or stroke, autonomic control of respiration may be compromised (1). Though mechanical ventilation has a prominent role as a lifesaving tool in the intensive care unit and for acute respiratory assistance, as little as 18 h of diaphragm inactivity and mechanical ventilation can result in its atrophy (2). Electrical stimulation of the phrenic nerve or the diaphragm has been considered as an alternative to mechanical ventilation both in the acute setting and for long-term use (3–7). Contrary to the natural muscle fiber recruitment order during muscle contraction, direct electrical stimulation of the nerves or intramuscular simulation of the neuromuscular junction results in recruitment of muscle fibers in the reverse order, recruiting larger highly excitable fast fatigable fibers first, followed by smaller slower fatigue resistant fibers (8). The rate of activation of fiber units during functional electrical stimulation is also much higher (25–75 Hz) compared to voluntary fiber unit recruitment (5–10 Hz) (9). Thus, because of both reverse recruitment and the increase in fiber unit recruitment rate, electrical stimulation can result in muscle fatigue at a faster rate than under natural conditions.

In order to maintain sufficient muscle contraction to maintain a desired functional outcome, e.g., breath volume for adequate ventilation, with the onset of fatigue, the electrical stimulation intensity would have to be adjusted. A closed-loop adaptive control system could compensate for the fatigue by increasing stimulation without requiring constant manual tuning. However commercially available pacing systems do not offer this ability. Also, during diaphragm muscle pacing alone, diaphragmatic contraction causes inward movement of the rib cage due to inactivity of the intercostal muscles, reducing the intrathoracic volume. This reduction in volume diminishes the efficiency of diaphragm stimulation, leading to a lower tidal breath volume (10). Canine studies have indicated that combining upper thoracic stimulation in conjunction with diaphragmatic stimulation can ameliorate this and elicit a tidal volume greater than the sum of the volume elicited by stimulating each respiratory muscle alone (10, 11).

Another feature that is unaccounted for is the ability to induce periodic sighs. Sighing has profound physiological and psychological benefits (12, 13). An adult human being sighs approximately once every 5 min (12). Sighs can reset regular breathing, improve gas exchange, and prevent the progressive collapse of alveoli by expanding them during a long, deep breath (14). In acute respiratory failure patients, mechanical ventilation incorporated with periodic sighs can decrease lung strain, ventilation heterogeneity, and increased gas exchange (13, 15), but the shortcomings associated with mechanical ventilation, such as the risk of muscle atrophy, impairment in mobility, and the necessity for tracheal intubation remain.

We recently developed an adaptive closed-loop intramuscular diaphragm (*Dia*) pacing system that can adapt the stimulation parameter to automatically personalize stimulation patterns and adapt to achieve adequate ventilation for meeting metabolic needs (16, 17). To delay the onset of diaphragm muscle fatigue during long-term pacing and include sigh-like augmented breaths periodically, we have expanded the capabilities of the system by the addition of external intercostal (*EIC*) muscle stimulation that supports thoracic stabilization and expansion of the upper rib cage to prevent inward thoracic movement. Adding adaptive external intercostal stimulation could lower the stimulation charge required for diaphragm contraction to attain the desired tidal breath volume, thus delaying stimulation-induced fatigue of the diaphragm. Additionally, sighs could be induced with lower charge delivery to the diaphragm.

Thus, we have introduced a novel approach to personalized pacing that can counteract stimulation-induced diaphragmatic fatigue during respiratory pacing, induce sigh-like behavior, and that has the potential to adapt to changes in metabolic needs. Such a control system offers the ability to significantly enhance the use of respiratory pacing systems for weaning patients from mechanical ventilation and for long-term chronic use.

## Materials and methods

### Study design

Experiments were conducted on *n* = 8 anesthetized, spontaneously breathing, adult male Sprague Dawley rats weighing 400 ± 80 g. Rats were maintained under anesthesia with subcutaneous urethane (50 mg/kg) injection followed by supplementary isoflurane (0.5%–3%) inhalation. Toe pinch reflex and respiratory rate were used as an indicator of the proper level of anesthesia. The body temperature was maintained around 37°C with the help of a closed-loop thermal pad (TCAT-2DF controller, Physitemp instruments Inc., NJ, USA) and a rectal thermometer. 30G stainless steel electrodes were inserted subcutaneously in chest muscles to record the electrocardiogram and monitor heart rate. A chest-mounted respiratory belt was utilized to monitor the breathing pattern and breathing rate of the animal. To avoid dehydration, a lactated ringer solution was injected subcutaneously every 2–3 h.

### Ethics statement

Rats were housed individually in the university animal care facility with a 12-hour light/dark (reverse with natural cycle) cycle with food and water *ad libitum*. All the procedures were approved and are in accordance with the guidelines established by the Institutional Animal Care and Use Committee at Florida International University.

### Tracheostomy and intramuscular electrode implantation in the diaphragm, external intercostal muscle

After tracheostomy and placement of a custom tracheal tube, a small pneumotachometer (PTM type HSE-73-0980) was directly connected to the tracheal tube to measure airflow. This flow was integrated (PI-1000, CWE Inc. Ardmore, PA, time constant = 0.2 s) to obtain the breath volume on a breath-by-breath basis. A capnograph (CapStar-100, CWE Inc., Ardmore, PA) measured end-tidal CO_2_ (etCO_2_) throughout the experiment to ensure normocapnic etCO_2_ (35–45 mmHg) was maintained. To implant pacing electrodes in the diaphragm muscle, a 2–2.5 cm incision along the linea alba was made caudal to the xiphoid process. A hand-operated stimulator (DigiStim 3 plus, Neuro Technologies), providing a single monophasic pulse at 1 Hz, was used to map the motor point location in each hemidiaphragm. Custom-made stainless-steel wire electrodes [Diameter 0.002″ (bare), 0.0045″ (coated), with a 2–3 mm active region] were implanted bilaterally approximately 1 mm from the point of stimulation that elicited the strongest diaphragm contraction. A study in the adult canine model showed that the most substantial increase in inspiratory volume could be induced by pacing 2nd intercostal spaces compared to stimulation of other intercostal spaces (10). Hence, after an incision was made along the midline of the chest, muscle layers (trapezius and latissimus dorsi) were removed by blunt dissection to identify the 2nd intercostal space and the external intercostal muscles exposed. The hand-operated stimulator was used to map the muscles and bilateral electrodes were implanted intramuscularly.

### Diaphragm, external intercostal muscle pacing

**Figure 1:**
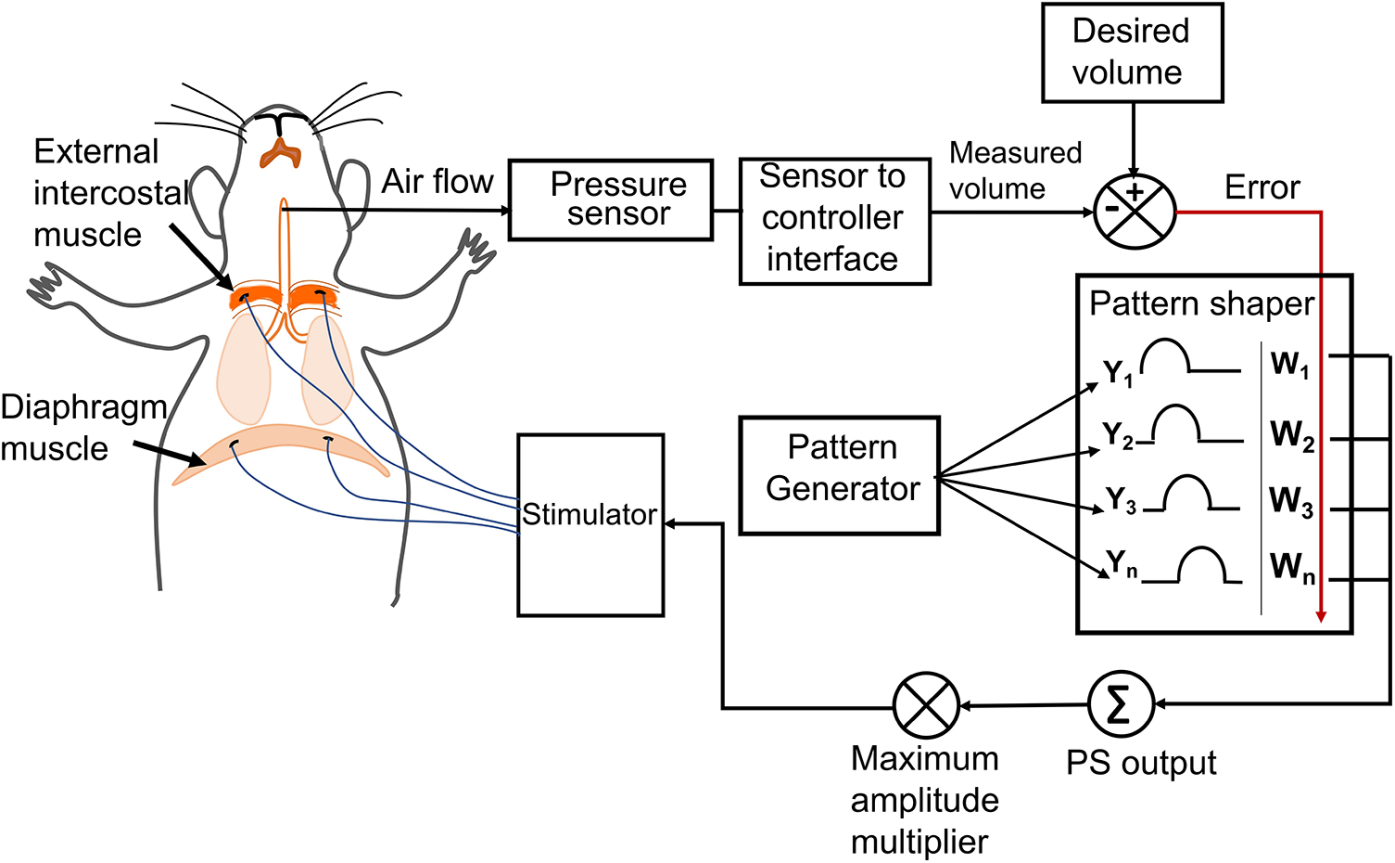
The adaptive neuromorphic controller architecture. Electrodes were implanted bilaterally in the diaphragm and external intercostal muscles and controlled using an adaptive controller following a pattern generator (PG)/pattern shaper (PS) scheme. The PG is a fixed oscillator that generates a fixed breath duration (cycle period) for each breath. The PS is an artificial neural network that adapts stimulation output (current amplitude) to all muscles based on the instantaneous error between the desired and measured breath volume. The maximum allowed current amplitude for stimulating each muscle is independent and accounts for differences in individual muscle activation properties and electrode impedances.

The twitch threshold current (the minimum current required to elicit visible muscle contraction) was determined for each electrode. Stimulation pulses were sent using a programmable constant-current stimulator (FNS-16, CWE Inc., Ardmore, PA) delivering cathodic first, biphasic current pulses of 80 μs/phase at 75 Hz (18), varying the current amplitude. The maximum current amplitude was set as 1.5–2 times the twitch threshold of the muscle, resulting in a stimulation current limit of 2–4 mA. The stimulation amplitude was determined by the adaptive controller whereas the twitch threshold facilitated us to set the maximum allowed amplitude for each muscle during the stimulation. Adaptive pacing used the same stimulation parameters as described above. The previously designed controller's capability [developed by Siu et al. (16)] was expanded by adding two independent stimulation channels while keeping the original configuration consisting of a pattern generator for setting the respiratory rate and a pattern shaper to set the breath volume pattern same (Figure 1). The single Pattern Shaper thus allowed simultaneous adaptive control of the stimulation of the diaphragm and the external intercostal muscles.

### Experimental protocol

The adaptive controller modulates the stimulation parameters to match the desired breath volume on a breath-by-breath basis with a fixed breath duration (cycle period). Each trial consisted of at least 1 min of spontaneous breathing, followed by at least 5 min and at best 15 min of adaptive stimulation. For each trial, the desired breath volume pattern and cycle period during pacing were obtained by averaging the pre-stimulation recorded breath cycles except for non-breathing behavior cycles, i.e., sighs. As intact rats have an intrinsic breathing pattern, successful entrainment of the stimulation-assisted breath with the intrinsic breath requires the stimulation-assisted breath to have a larger tidal volume. Hence, the desired breath volume needed was set at an *ad-hoc* 120% of the baseline volume (16). Experimental trials were spaced 20–30 min apart in order to allow the inspiratory muscles to recover from stimulation-induced fatigue. We recorded one trial for each type of stimulation and the order of the trials was randomized across animals.

**Figure 2:**
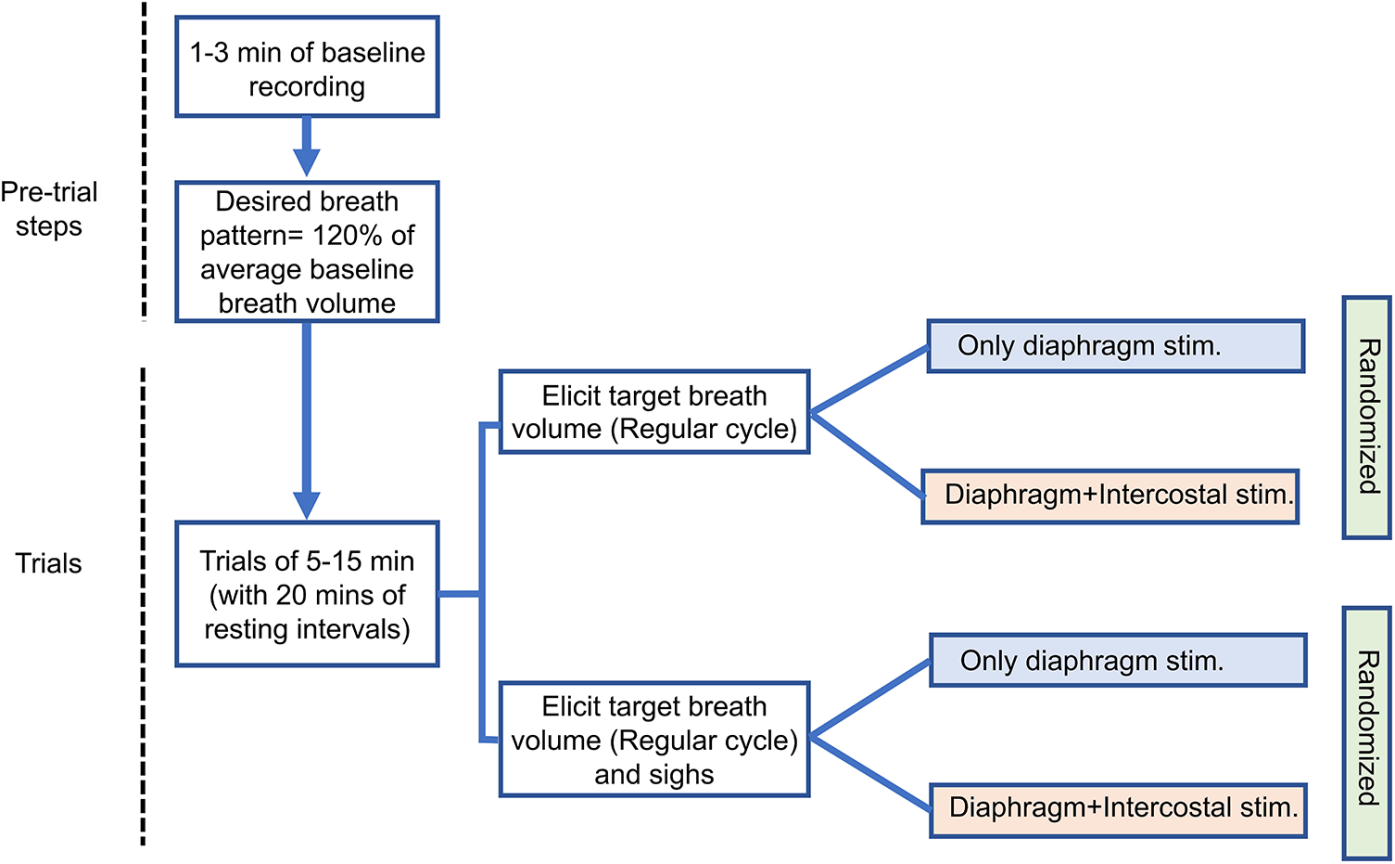
Experimental sequence diagram. The arrows indicate consecutive steps of experiment. One trial for each stimulation type was recorded. Separate color schemes have been used for different stimulation approaches.

We performed a separate set of trials for eliciting a targeted breath volume pattern with sighs (Figure 2). The evoked sigh was set to occur after every 30 breaths cycles. For inducing sighs, the controller stored the PS outputs (time-shifted weighted summation of neuronal output) of each update for the previous breath cycle and sent out twice the magnitude of PS output to the controller. The adaptive learning in the controller was paused automatically every 30 cycles, during the insertion of the sigh and the following cycle. The doubling of the amplitude of stimulation on each time step (every 40 ms) caused the inspiratory muscles to contract by an additional degree.

### Strength-duration curve determination

In order to assess the optimal pulse width for stimulation, strength duration (SD) curves were generated by plotting the twitch threshold current required (strength levels) versus the pulse widths (duration of the stimulating pulse). The stimulation frequency was kept the same for both diaphragm and external intercostal muscle pacing. Decreasing pulse widths from 500 to 100 µs in steps of 100 µs and 100 µs to 10 µs in steps of 10 µs were used (8). We allowed a 60s rest period between consecutive pulse stimuli to prevent carryover effects of the stimulation on muscle recruitment. In general, from the SD curves, we determined the rheobase (twitch threshold current at infinite pulse duration; here 500 µs) and a range of chronaxie (stimulus duration at the point where the twitch threshold current is twice the rheobase) in order to assess the excitability of the muscle.

### The PG/PS controller

The controller was originally developed by Abbas and Chizeck (19), used in an incomplete spinal cord injury (iSCI) rodent model for cyclic limb movement (18), and in an iSCI rodent model for functional stimulation of the diaphragm muscle to attain a desired breath volume pattern (16, 17). The controller is a neural network system that can provide automated customization of the stimulation parameters to drive the desired action. It has two components: a pattern generator (PG) and a pattern shaper (PS). The PG has a pattern-generating capability based on neurophysiological models and sets the timing of the breath. In this study, a frequency oscillator was used to produce a fixed respiratory frequency, with a fixed period (16). The PS is a single-layer adaptive artificial neural network that modulates the current amplitude to elicit a desired breath volume trajectory. This adaptation is driven by the instantaneous error across the breath cycle between the measured breath volume and the desired breath volume. For a more detailed description of the PG/PS controller please refer to Siu et al. (16). For our experiments, one PS unit was used to stimulate both sets of muscles (Diaphragm and external intercostal) with the output to each muscle being modified by an independent gain factor determined via twitch threshold as described above.

### Performance measures

To evaluate performance of the controller, a percent inspiratory root-mean-squared error was calculated based on the desired breath volume and the measured breath volume at a given instant (16). Charge delivered per cycle was calculated as the summation of the product of the width and amplitude of each pulse sent during one cycle. We conducted a Wilcoxon signed rank test comparing the average charge delivery per cycle to the diaphragm muscles among the animals for both *Dia* and *Dia + EIC* stimulation. As electrical stimulation or the charge delivered to the muscle is responsible for fatigability, a metric for measuring fatigability, fatigue index (FI) was measured that represents relative changes in charge delivered to the muscle. Fatigue index for each trial was calculated as the difference in the average initial charge delivered per cycle and final charge delivered per cycle, normalized by the initial charge per cycle (18). As muscles get fatigued during the time course of trials, higher level of charge is delivered during stimulation to maintain the same functional level. This relative increase in charge delivery or fatigue index was used as a measure of muscle fatigability during the stimulation in a given trial. For calculating the fatigue index, we chose the initial and final cycle sets, each consisting of 50 cycles and approximately 400 cycles apart. These sets were considered in such a way that there is a minimal number of cycles without entrainment. We considered the initial set of cycles when the measured breath volume and desired breath volume started to achieve entrainment with a minimal phase difference. The initial charge per cycle set and the final charge per cycle set was approximately 400 cycles apart. For animal 1 and animal 2, the number of available cycles for analysis was not sufficient, hence the fatigue index was not calculated for these animals. Thus, the fatigue index analysis includes *n* = 6 animals.

Since the desired tidal volume varied among all animals and sometimes for different trials for the same rat, we normalized the sigh tidal volume with the targeted tidal volume for each trial. We denoted this normalized value as the “sigh tidal volume factor.” For animal 7, only trials for inducing regular breath patterns could be recorded, but trials for inducing sighs could not be recorded and were not included in the data analysis. Thus, the sigh tidal volume data analysis included *n* = 7 animals.

### Data acquisition and data analysis

Each recording trial was 5–15 min long, consisting of at least 60 s of intrinsic breathing, followed by stimulation-augmented breathing. At least a 20–30-min rest period was allowed between trials. Data recording included EKG, breath volume, charge delivered, and PS output (Normalized simulation output). All the data from the PG/PS controller were collected at 25 Hz, and other recorded data were collected at 10 kHz, which later was down-sampled to 25 Hz. A one-way Wilcoxon signed rank analysis, that assumes non-normal distribution of the dataset and accounts for small sample sizes, was conducted in SPSS on fatigue index data with *α* = 0.05 significance level to test if the charge delivered per cycle to the diaphragm muscle was significantly lower with *Dia + EIC* muscle stimulation. Besides, we performed one way Wilcoxon signed rank test on the average charge delivered per cycle across the animals for *Dia* and *Dia + EIC* stimulation trials. The tidal volume factor of sighs was averaged across all the sighs for each observation for *Dia + EIC* muscle stimulation and *Dia* muscle stimulation separately. The one-way Wilcoxon signed-rank test was performed on the averaged tidal volume factor for *Dia* and *Dia + EIC* to test if tidal volume generated for the sighs was significantly larger in *Dia + EIC* muscle stimulation than *Dia* muscle stimulation.

## Results

Overall, the following sections present data from *n* = 8 animals. In 2 animals the trials were short (5 min long) therefore for the fatigue index analysis, data from *n* = 6 animals were included in the analysis. We could not run the trials for inducing sigh in one animal. Hence, data from *n* = 7 animals were included for the sigh tidal volume analysis. Our results indicate that adding adaptive external intercostal stimulation could lower stimulation required by the diaphragm to attain the desired tidal breath volume, thus delaying stimulation-induced fatigue of the diaphragm. Besides the addition of external intercostal muscle pacing could facilitate induction of periodic augmented breaths. The following sub-sections report details.

### Determination of pulse width of stimulation by strength-duration curve parameters

**Figure 3:**
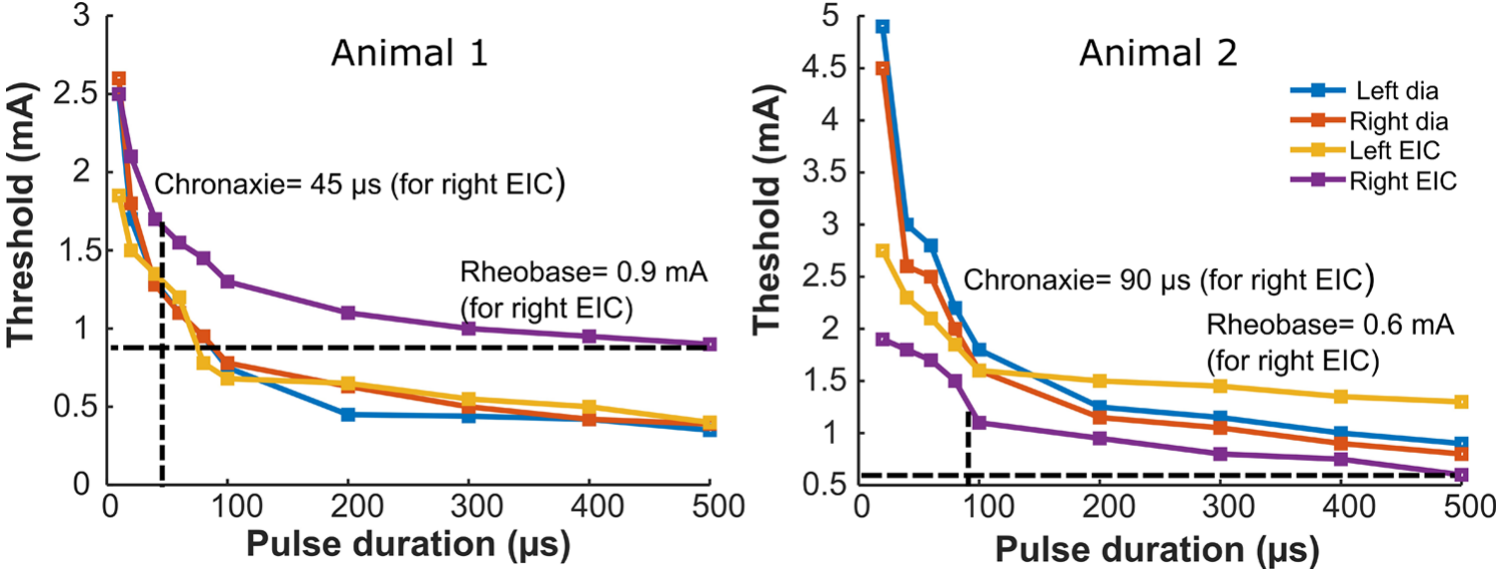
Strength-duration curves for bilateral stimulation of the diaphragm and external intercostal muscles. The panels show strength duration curves for two different animals. Rheobase refers to the lower limit of stimulus intensity needed to excite the muscle fiber at a very long stimulus duration (500 µs here). Chronaxie refers to pulse duration at which the threshold is twice the rheobase value. Strength duration curves for stimulation of the left or right diaphragm, or the left or right external intercostal muscles are shown for two animals. Also illustrated are horizontal dashed lines that identify the rheobase value and vertical dashed lines that identify the chronaxie for the Right EIC Strength duration curves; blue = Dia left, orange = Dia right, yellow = EIC left, violet = EIC right.

Strength duration curves were generated by plotting the minimum intensity (amplitude) of an electrical stimulus required to produce a twitch with different pulse width durations up to 500 µs. Figure 3 shows a nonlinear hyperbolic relationship between the minimum stimulus strength at each pulse width of stimulation. The optimum stimulus pulse width was defined by the chronaxie value determined from the SD curves. For all four muscles of both the animals, rheobase value ranged from 0.35 to 1.3 mA, and the chronaxie values ranged from 35 to 100 µs. The average chronaxie value across all the muscles for both animals were calculated to be 80.75 ± 27.52 µs. Based on this calculated chronaxie guideline, we used 80 µs as our stimulation pulse width.

### Adaptive closed-loop control can achieve desired tidal volume pattern

**Figure 4:**
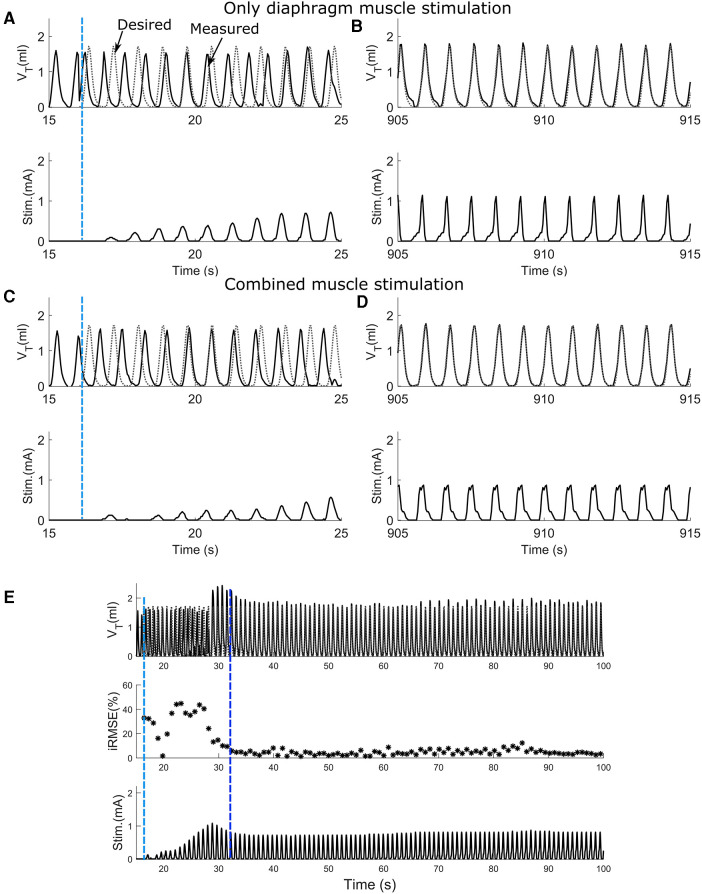
The ability of the adaptive PS controller to assist attaining the desired breath volume pattern in intact animal during diaphragm muscle stimulation or combined muscle stimulation. (**A,C**) Response to adaptive closed-loop control of diaphragm muscle stimulation or combined muscle stimulation respectively in a rat. Stimulation output progressively increases to counteract the mismatch between the measured stimulation assisted breath volume trajectories (solid line) and the desired breath volume trajectory (dotted line). The vertical light blue dashed line shows when the controller was turned on. (**B,D**) Continuation of **A** and **C** respectively showing the last 10 s of the trial for diaphragm muscle stimulation and combined muscle stimulation. In both cases, the stimulation assisted breath volume pattern overlaps the desired breath volume pattern. (**E**) The adaptive closed-loop controller implementation for the combined muscle stimulation (same animal, same trial as **C,D**) for first 100 s. In the beginning, iRMSE was high; to match the stimulation elicited instantaneous volume with the desired instantaneous volume, stimulation output adapted and initially increased cycle by cycle. The second vertical dark blue dashed line shows where the stimulation assisted breath volume started to match the desired volume. iRMSE value decreased to <10%, and the stimulation output remained stable for the duration of the trial.

To assess the effect of synergistic muscle activation by respiratory pacing, trials in which only the diaphragm was stimulated and trials in which both the diaphragm and 2nd intercostal muscles were stimulated were carried out. Figure 4 shows the outcome during the stimulation of respiratory muscles with the adaptive closed-loop controller. The stimulation output initially starts at zero when the controller is turned on for the diaphragm-only stimulation (Figure 4A), and combined muscle stimulation (Figure 4C). The controller adapts stimulation output to match the stimulation assisted breath volume pattern with the desired breath volume pattern by increasing the charge delivered. After approx. 15 min of pacing, the assisted breath volume pattern continues to match the desired breath volume pattern for both the diaphragm-only stimulation (Figure 4B) and combined muscle stimulation (Figure 4D). Figure 4E shows an example of the controller's pattern-shaping capability for the first 100 s of a 15-minute-long trial for combined muscle stimulation. The vertical light blue dashed line at the beginning of the trial indicates turning on of the controller. The inspiratory root mean square error (iRMSE) value increases to account for the difference in volume between the stimulation-assisted breath and the desired breath. Based on the error value in each instance, the stimulation amplitude adapts to match the desired breathing pattern. Once the stimulation-assisted breath volume pattern starts to match the desired breathing pattern, iRMSE, as well as the stimulation output, starts decreasing. For our investigations, when the iRMSE value decreased to 10% or less and remained there for at least 20 pacing cycles, the controller was considered to have “adapted,” which is indicated in Figure 4E by the second vertical dark blue dashed line.

### Combined muscle stimulation reduces diaphragmatic fatigue

**Figure 5:**
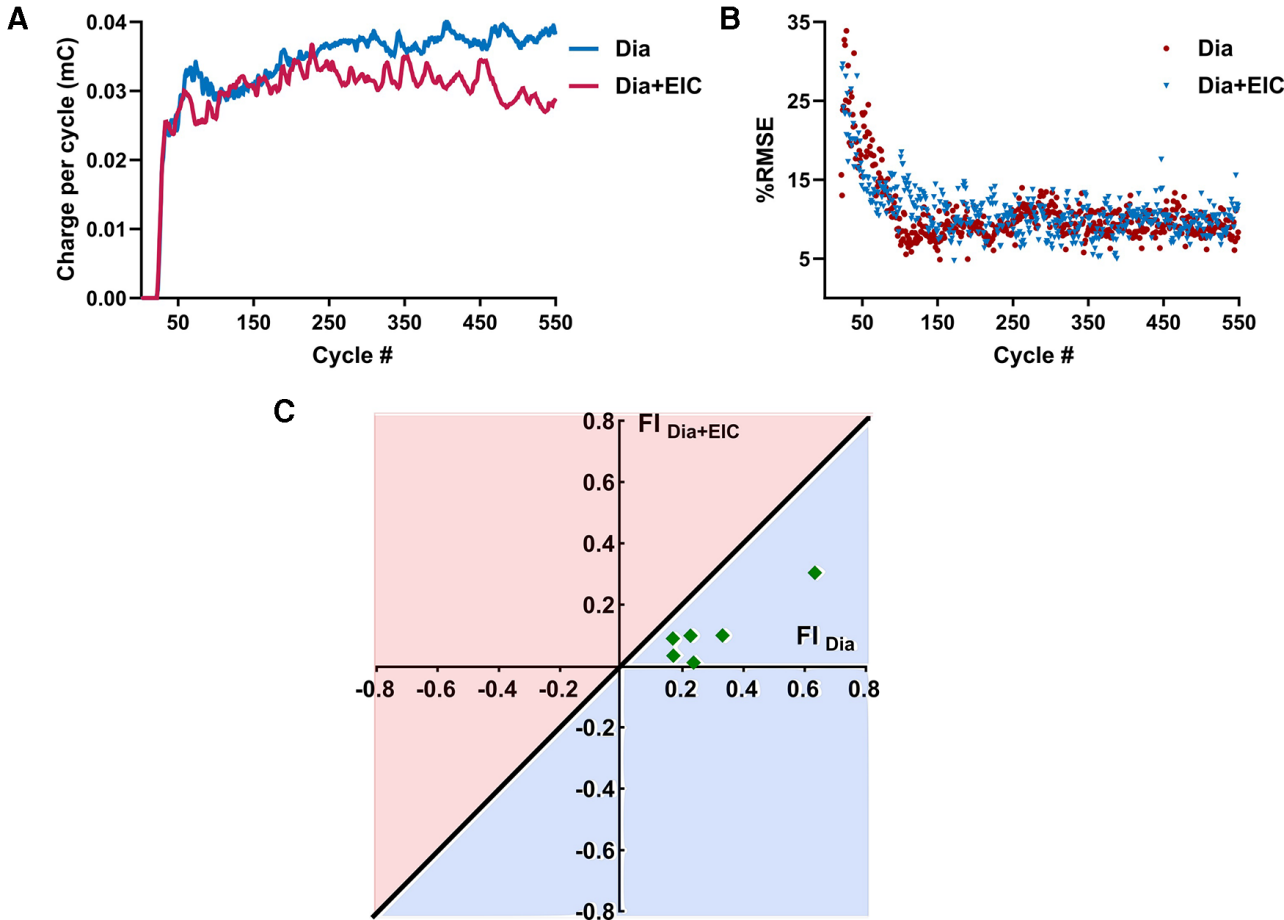
Averaged charge delivered per cycle to the diaphragm muscle and averaged %iRMSE over the animals and fatigue index representation during combined muscle pacing and diaphragm pacing. (**A**) Averaged charge delivered per cycle to the diaphragm muscle for diaphragm-only muscle stimulation (in blue) and combined muscle stimulation (in red). (**B**) Averaged %iRMSE over the breath cycles of six animals for diaphragm muscle stimulation alone (in blue) or combined muscle stimulation (in red). At the beginning of a trial, %iRMSE was high, but once the desired volume trajectory was obtained, the %iRMSE value decreased. (**C**) Fatigue index for diaphragm-only muscle stimulation (FI_Dia_) and combined muscle stimulation (FI_Dia + Eic_). The diagonal line going through origin presents the ratio of FI = 1; any point below the line (the light blue area) suggests less fatigue susceptibility and the points above the line (the light red area) suggests greater fatigue susceptibility of the diaphragm muscle to stimulation.

Figure 5A shows the average charge per cycle delivered (mC) to the diaphragm muscle during *Dia* stimulation (in blue) or *Dia + EIC* stimulation (in red) across all animals for the first 550 cycles. These traces show that charge delivered per cycle to diaphragm muscle increases in the later cycles of the trial (cycles 300–550), suggesting that to attain the desired volume pattern more charge is required to maintain a similar breath volume. One way Wilcoxon signed rank test showed that the average charge per cycle delivered to the diaphragm muscle during *Dia + EIC* stimulation was significantly lower compared to *Dia* stimulation (*p* < 0.001). The decrease in charge delivered per cycle to attain the desired volume pattern indicates that the diaphragm muscle's susceptibility to fatigue decreases when paired with intercostal stimulation, thus the results suggest that *Dia + EIC* stimulation leads to a slower onset of fatigue. Figure 5B shows %iRMSE averaged over the six animals for the first 550 cycles of *Dia* stimulated and *Dia + EIC* muscle stimulated trials (blue and red in color, respectively). It shows that initially, %iRMSE was high, but once the controller facilitates matching the desired breathing pattern, %iRMSE value decreased. Although, because of occasional loss of entrainment, the %iRMSE cycled between a high and low value from one breath to the next. In order to assess whether combined muscle stimulation facilitates faster entrainment and better alignment between the stimulation-induced breath volume and the desired breath outcome compared to single muscle stimulation, we quantified the number of breath cycles required to achieve entrainment. For the analysis, we set *ad-hoc* criteria for the iRMSE to be less than 20% for at least 20 breaths. There was one animal who didn't achieve entrainment for *Dia* stimulation and another animal who didn't achieve entrainment for *Dia + EIC* stimulation, these two animals were excluded from the analysis. Thus, we conducted statistical analysis to determine the number of cycles needed to achieve entrainment for each condition for the remaining 6 animals. A Wilcoxon signed rank test showed that in combined muscle stimulation, number of cycles required to achieve entrainment were significantly lower than for diaphragm stimulation [51 ± 20 vs. 114 ± 47 cycles needed (average ± standard error) to achieve entrainment, *p* = 0.04].

Figure 5C shows the fatigue index (FI) plotting for the diaphragm muscle during diaphragm muscle stimulation and combined muscle stimulation for six animals. The diagonal blue line through the origin (0, 0) represents the line where the fatigue index is the same for the diaphragm-only stimulation (FI_dia_) or combined stimulation of the diaphragm and external intercostal (FI_Dia + Eic_). A positive fatigue index indicates that greater charge delivery was required during the latter part of the trials, while a negative fatigue index indicates that a lower charge delivery was required for the latter part. The region under the straight line represents the area where the fatigue index was higher when only the diaphragm muscle was stimulated. Figure 5C illustrates that all the data was under the diagonal line, which suggests that the diaphragm muscle was more fatigued during diaphragm-only muscle stimulation than during combined muscle stimulation. The statistical test showed that FI values of the diaphragm muscle were significantly lower during combined muscle stimulation (*p* = 0.014).

### Pacing can be used to elicit sigh-like behavior

**Figure 6:**
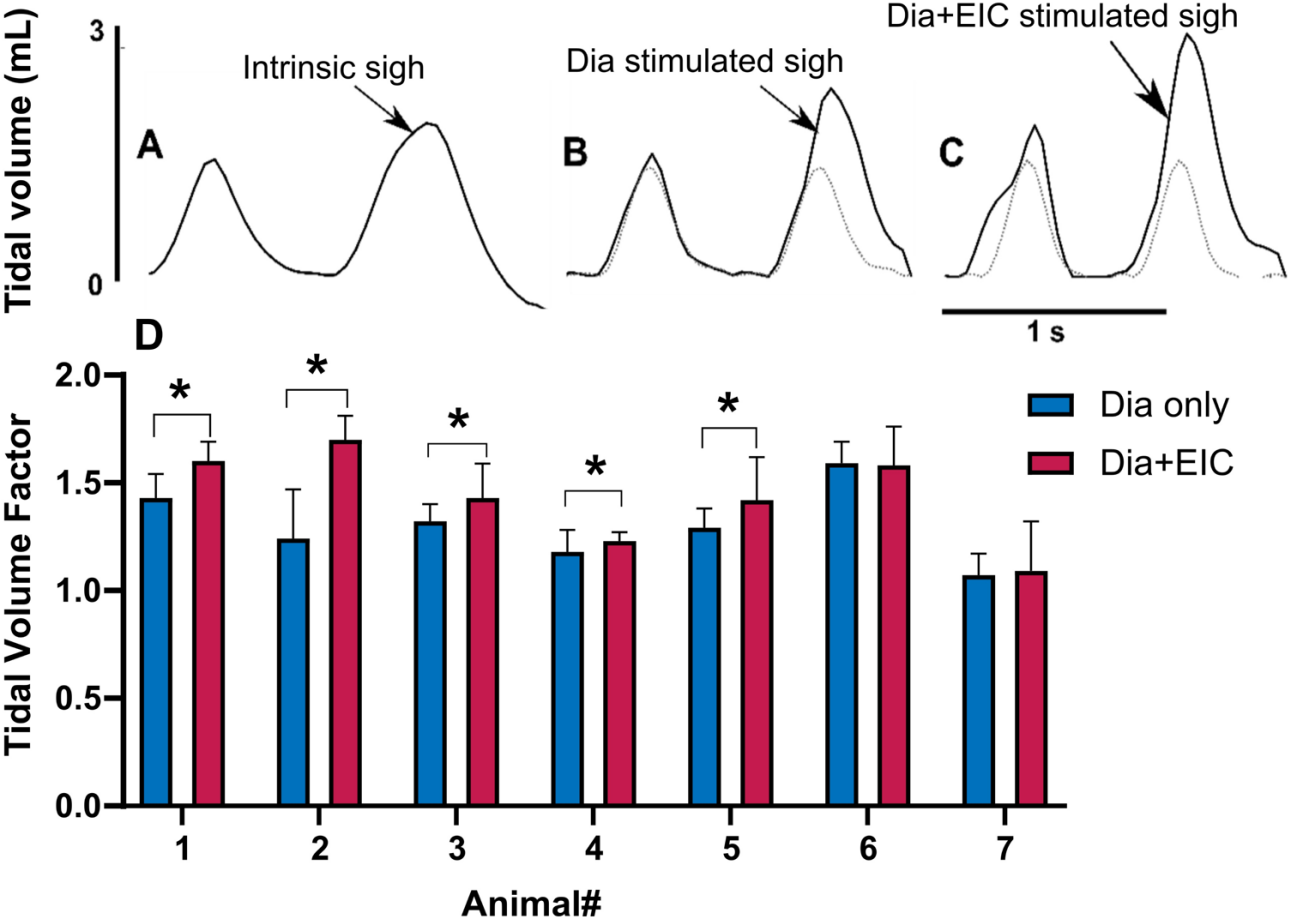
Eliciting sigh-like behavior by diaphragm muscle stimulation and combined muscle stimulation. Representative breath volume traces showing a regular breath followed by an intrinsic sigh during spontaneous breathing (**A**), a paced sigh using diaphragm-only stimulation (**B**), and a paced sigh using combined muscle stimulation (**C**). (**D**) Average tidal volume factors for sigh in diaphragm-only muscle stimulation (in blue) and combined muscle stimulation (in red) for all the animals, where *indicated *p* < 0.05.

To assess the controller's ability to induce sighs, we performed *Dia* muscle stimulation and *Dia + EIC* muscle stimulation separately as in the previously described trials, however, stimulation at twice the value of the previous cycle was delivered every 30 cycles to induce augmented breaths, or sighs. Figures 6A–C shows intrinsic sighs, sighs induced with diaphragm-only stimulation, and sighs induced via combined stimulation, each compared to the previous non-augmented breath. Figure 6D shows the sigh tidal volume factor (measured sigh tidal volume/desired non-sigh tidal volume) obtained during diaphragm-only muscle stimulation (in blue) and combined muscle stimulation (in red) for seven rats. Even though the trial duration was the same during *Dia + EIC* or *Dia* stimulation, the number of sighs could be different because of different respiratory rates. For the mean ± SD calculation, within one animal, the smaller number of sighs between the two trials (*Dia + EIC* or *Dia*) was utilized. One-tailed paired sample *t*-test for within subject average tidal volume factors shows that in 5 out of 7 cases, average tidal volume factors were significantly larger in combined muscle stimulation than in diaphragm-only stimulation. One way Wilcoxon signed rank test for between subject average tidal volume factor indicated that the volume factor for combined muscle pacing was significantly higher than that for diaphragm pacing -only (*p* = 0.014).

## Elicitation of sighs can reset synchronous entrainment

**Figure 7:**
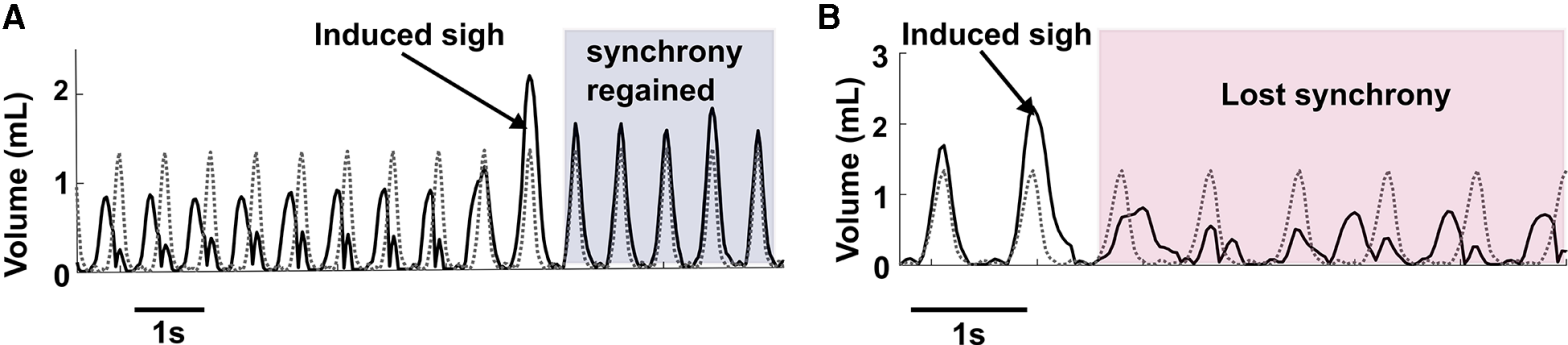
Elicitation of sigh can reset synchrony. (**A**) Shows an example of sigh functioning as a re-setter to restore synchrony between desired breathing and induced breathing. (**B**) Represents an example where after a sigh elicitation, loss of synchrony occurred between the desired and induced breath pattern.

Figure 7A illustrates the ability of sighs to reset synchronous entrainment when the intrinsic breathing pattern and that elicited by inspiratory stimulation were not synchronous. If there were at least 3 breaths where the measured and desired breath initiation was out of phase, we considered that as loss of synchrony before a sigh. During stimulation assisted breathing, sighs aided in the alignment and synchronization between the desired and measured tidal volume patterns. In our experiments, in 12 sighs during different trials in all animals, we observed 11 sighs (91%) reset the alignment.

While we were able to consistently induce sighs, we observed rare instances of loss of synchrony after the induced sigh (Figure 7B) during combined muscle stimulation. As combined muscle stimulation induced a larger sigh volume compared to single muscle stimulation, the expiration (downwards phase in a breath cycle) takes longer to complete than the desired expiration, resulting in a phase mismatch between the volume pattern of the induced and desired breaths. Synchrony took several breaths to reoccur, in some cases lasting until the following induced sigh.

## Discussion

This investigation presents the viability of a novel approach to ventilatory pacing by simultaneous diaphragm and external intercostal muscle stimulation using an adaptive closed-loop controller to achieve a desired respiratory breath pattern including sighs. We showed that it is possible to attain a targeted breath volume pattern using this synergistic approach of combined muscle stimulation, that the target could be reached earlier, that this strategy leads to a reduction in diaphragmatic fatigue onset, and that the strategy allows for inclusion of efficient sigh-like augmented breaths within the breathing pattern. Combined, these factors could lead to improved respiratory health in individuals that depend on diaphragmatic pacing.

Diaphragmatic fatigue can occur due to electrical stimulation and hence a need to modulate the stimulation parameters to achieve the desired effect on ventilation has previously been identified (20). Besides changing stimulation parameters to overcome diaphragmatic fatigue, one can also activate additional inspiratory muscles to attain the desired ventilation, thereby reducing the need for stronger diaphragmatic contractions. Indeed, when we combined diaphragmatic and external intercostal muscle stimulation and used the adaptive controller, we found that the fatigue index of the diaphragm muscle calculated during the long trials (>550 cycles) of stimulation was significantly lower during combined muscle stimulation while also being able to achieve the desired volume pattern. The lower charge delivery utilized for diaphragmatic contraction likely resulted in fewer diaphragm muscle fibers being recruited by electrical stimulation and hence reduced overall diaphragm muscle fatigue. Presumably, the contraction of the external intercostal muscle allowed the expansion and stabilization of the upper rib cage during the combined muscle stimulation. The additional expansion of the upper rib cage increases the volume of the thoracic cavity and decreases intra-alveolar pressure and more air is drawn into the lungs. Thus, the external intercostal muscle pacing facilitates inhalation and enhances respiratory mechanics, thereby reducing the need for higher charge delivery to the diaphragm to achieve the desired volume. Hence, this approach can be used to reduce stimulation-induced fatigue of the diaphragm muscle.

Since the animals were anesthetized with isoflurane, a respiratory depressant, the respiratory rate decreases and results in an elevation of the PaCO_2_. The increased breath volume not only assists in triggering the Herring-Breuer reflex and promoting entrainment, but also alleviates the increased PaCO_2_ that results from slower breathing. During this study, we did not observe significant deviations from normative end-tidal CO_2_ (etCO_2_) values, and thus did not study the relationship between etCO_2_ and stimulation parameters. The second aim of our investigation was to assess the closed-loop controller's ability to generate sigh-like behavior by diaphragm-only or combined diaphragm and external intercostal muscle stimulation. Currently, some mechanical ventilation systems incorporate periodic sighs for the re-aeration of collapsed alveoli (15). However, the shortcomings of mechanical ventilation persist. Aside from mechanical ventilation, available phrenic nerve pacing systems can also be used to provide sigh breaths by an intermittent increase of stimulus frequency (5). However, since all the phrenic nerve pacing systems are open-loop systems, manual adjustment of stimulus parameters is needed for inducing sighs. Our results indicate that combined muscle stimulation can produce a larger tidal volume than diaphragm-only muscle stimulation. Periodic breaths with larger volume can help inflate more alveoli and thus might prevent atelectasis (13, 21). Following the sigh, the adaptive controller adjusted the stimulation parameters automatically to return the ventilatory pattern to the desired ventilatory pattern (5, 15). Thus, our approach provided an automatic cyclic increase in stimulation amplitude to elicit a large tidal volume without the need for human intervention.

Another benefit of including periodic sighs into the pacing paradigm is related to the entrainment of the paced breaths to the intrinsic breaths. When diaphragmatic pacing at a fixed cycle period is implemented over an underlying spontaneous breathing rhythm then the two rhythms must entrain themselves; introduction of sighing was found to improve this synchrony. When a loss of synchrony between the intrinsic breathing and stimulation assisted breathing occurred before a sigh, the sigh helped in resetting the intrinsic breathing pattern and aligning the desired and measured volume patterns. Previous studies also indicate that sighs function as a re-setter for intrinsic breathing by restoring lung resistance and compliance back to a normal level (21). The intrinsic central pattern generator gets feedback during sighing since the large breath during a sigh activates pulmonary stretch receptors. While we were able to induce augmented breaths, we did observe instances of loss of synchrony after the induced sigh. This occurred possibly due to the shorter expiratory time in the induced sigh compared to the intrinsic sighs. It may be possible to prevent this loss of synchrony by extending the duration of the sigh, as entrainment with mechanical ventilators has been associated with longer breaths with lower flow rates and higher volumes (22). Further experiments are required to assess if post sigh loss of synchrony is observed in animal models that have impaired breathing such as after incomplete spinal cord injury, and if a longer induced sigh duration would be beneficial for re-synchronization.

Our empirical study in an animal model showed that the synergistic approach of combined muscle stimulation of multiple inspiratory muscles can be used to reduce diaphragmatic fatigue onset, and for efficient sigh-like breath elicitation. During the implementation of diaphragm stimulation in clinical environments for partients, the range of stimulation frequencies typically spans from 20 Hz to a maximum of 50 Hz (23). However, a stimulus frequency of 20 Hz is often preferred. When diaphragm pacing is prolonged, it becomes necessary to readjust the stimulation parameters to prevent fatigue caused by stimulation. Typically, a decrease in stimulation frequency is employed as a preventive measure against fatigue since higher frequencies have the potential to induce muscle fatigue (24). Low-frequency stimulation has been found to enhance the endurance properties of electrically stimulated muscles by transforming the composition of muscle fibers from a mixture of types to a predominantly type I fiber population. However, this transformation also leads to a notable decrease in fiber diameter as well as decreases the capacity for maximum force generation (23). Consequently, the ability to generate maximum inspired volume is compromised.

In our study, to optimize the ability to generate maximum force, a stimulation frequency of 75 Hz was selected. Furthermore, to mitigate fatigue induced by stimulation, incorporating additional pacing to the external intercostal muscles has shown to be effective. This approach enables the generation of higher tidal volumes while reducing the amount of charge delivered to the diaphragm muscle. Besides, the inspiratory cycle tidal volume was augmented by muscle pacing, whereas expiration was passive. As a result, expiration-muscle-assisted behaviors like coughing that are essential for clearing the airways of mucus, dust and germs, cannot be influenced. Hence, an abdominal muscle stimulation paradigm could be an additional component to the controller. This additional component would allow pacing to offer a complete set of desired ventilatory behaviors that included regular breaths, periodic sighs, and user triggered coughs to assure good respiratory health.

The combined respiratory muscle pacing could be a promising option for ventilator-dependent individuals with spinal cord injury. In patients with a functional phrenic nerve, combined external intercostal and diaphragm pacing might maintain full-time ventilatory support. Abdominal muscle pacing would offer on-demand cough. The adaptive controller with multiple muscle pacing could also provide support by supplementing inspiratory volumes in individuals with partial phrenic nerve lesions. Clinical trials with human participants will be required to validate the closed-loop adaptive control for respiratory pacing and verify the benefits of this novel approach.

## Data Availability

The raw data supporting the conclusions of this article will be made available by the authors, without undue reservation.

## Author summary

Restoration of ventilation through respiratory muscle pacing has been shown to be a promising alternative to traditional mechanical ventilation. Though there are multiple muscles involved in our regular breathing, currently respiratory pacing only targets the diaphragm muscle. The pacing is associated with diaphragm muscle fatigue. Additionally, the pacing does not allow intermittent sighs like in natural breathing. Here, we present an adaptive respiratory pacing scheme that combines diaphragm pacing with the pacing of external intercostal muscles, which also contributes to inspiration. We investigate this system using a rodent model and demonstrate that the fatiguability of the diaphragm is significantly lower when the diaphragm and external intercostal muscle are paced together than diaphragm pacing alone. Besides, this multiple-muscle pacing scheme assists in the production of significantly larger sigh-like breaths. Our approach could be a promising option for long-term ventilation-dependent individuals to improve overall respiratory health by delaying respiratory muscle fatigue and introducing a sigh-like behavior.
